# Quality measurers of therapeutic communities for substance dependence: an international collaborative study survey in Latin America

**DOI:** 10.1186/s13011-017-0129-y

**Published:** 2017-12-20

**Authors:** Carlos Gómez-Restrepo, Patricia Maldonado, Nelcy Rodríguez, Rafael Ruiz-Gaviria, Miguel Ángel Escalante, Raúl Ángel Gómez, Marcelo Ribeiro de Araujo, Ana Carolina Schmidt de Oliveira, Joel Salvador Chávez Rivera, Jorge Alberto Godínez García, Marina Piazza Ferrand, Dora Blitchtein-Winicki

**Affiliations:** 1 Department of Clinical Epidemiology and Biostatistics, Hospital Universitario San Ignacio, Pontificia Universidad Javeriana, Bogota, Colombia; 2 School of Medicine, Pontificia Universidad Javeriana, Bogota, Colombia; 3 Unity of Epidemiological Studies in Mental Health, Psychology Faculty, National University of Cordoba, Cordoba, Argentina; 4 Department of Psychiatry, Federal University of São Paulo (UNIFESP), São Paulo, Brazil; 5 Consejero Terapéutico State Council Against Addictions, Guadalajara, Jalisco Mexico; 6 Polymetrix, Cabo San Lucas, Jalisco Mexico; 7 Universidad Peruana Cayetano Heredia, Lima, Perú; 8 National Institute of Public Health, Lima, Peru

**Keywords:** Therapeutic communities (TCs), Substance-related disorders, Patient satisfaction, Patient compliance

## Abstract

**Background:**

In Latin America, substance related disorders are highly prevalent and one of the treatment strategies is the Therapeutic Communities (TCs), however, in Latin America there is scarce data about this treatment strategies, their quality, drop-out rates and patient satisfaction.

**Methods:**

Based on a previous study in 5 Latin American countries, the TCs who had a score equal or higher than 9 according to the De Leon criteria which are some fundamental items that the TCs should meet, were selected to carry out a descriptive and retrospective study of qualitative and quantitative characteristics of the TCs.

**Results:**

Data from 58 TCs in 5 countries were included, with a sample of 1414 patients interviewed, of which most were single men, with no hospitalization history in a therapeutic community. Marijuana was the most commonly substance used in the 30 days prior to hospitalization, with 78% of interviewees referring alcohol consumption in the last 6 months and an average onset of psychoactive substances at 16 years of age. A 79% of the patients interviewed perceived some improvement during their stay in the TCs. The less fulfilled Quality Indicators by the TCs were “Requesting a professional qualification to former addicts that belonged to the program” and “Work as part of the therapeutic program”. Among the reasons for discharge found in the database, 44% were due to therapeutic discharge with fulfillment of the treatment plan and 44% withdraws.

**Conclusion:**

The user satisfaction with TCs, in terms of infrastructure and quality are quite high, as the fulfillment of essential quality items, however, the follow up information to evaluate effectiveness of the treatment is poor or in some cases unknown.

## Background

Use of psychoactive substances (PSs) is one of the psychiatric disorders that has generated great interest from governments because of the economic, political, social and health implications of their consumption [1–3]. Chronic use of these substances generates molecular and cellular changes that will effect in behavioral and lifestyle alterations that affect the individual suffering from such diseases [4]. In addition, use of PSs, is accompanied by increased risk behaviors, traffic accidents and co-infection with diseases like HIV and Hepatitis C virus [1, 5, 6].

In a study conducted by the World Health Organization (WHO)with several countries in different regions of the world, including Europe and the Americas, evaluating the prevalence of alcohol use disorders or other psychoactive substances in the past 12 months, a prevalence from 5.8% in Mexico to 13.2 in Germany [7] was found. The prevalence of such pathologies is not a negligible value taking into account the consumption of any substance, if we review that the substance use disorder accounted for 0.5 of the total years of life lost (YLL) worldwide, emphasizing that the most affected population is in the age group between 15 and 30 years [8] (6).

Nowadays the management and support of patients with substance use disorders are very wide but their objective is helping them to avoid use of the substance and abandon drug seeking behaviors. Despite the treatment and commitment of the patient to the management, research has shown relapse rates of up to 40 or 50%, so the treatment has to be done in more than one occasion [9].

One of the treatmen or management options are the TCs. These are self-help programs for abandonment of harmful substance use behaviours and health recovery of the patient through an individual personal growth, that is performed separating the subject from society and submitting the patients in a specific community with professional staff and other patients with substance use [10, 11].

TCs have proven to be effective in helping the patients abandon the active consumption of the substance, returning to their jobs, improving mental health and quality of life and also increasing the time to relapse and drug-free periods [12–15].

In the longest study conducted by the National Institue of Drug Abuse (NIDA), the DATOS study (Drug Abuse Treatment Outcome Studies) included more than 10,000 patients and compared the effectiveness of different therapeutic options for substance use, including TCs. The study showed that in those patients treated with this modality there was a reduction of heroin, cocaine and alcohol use after 1 and 5 years of fulfilling the treatment. Besides, this study also showed increased patient employment rates after the end of the treatment, proving through different outcomes measures the effectiveness of the TCs [16].Despite that some studies have shown the impact of the TCs, not all the TCs fulfil the quality standards recommended by the World Federation of Therapeutic Communities (WFTC). This variability can explain the different in the success and relapses rates [14].

Studies of the amount, quality and need of the TCs has been done mostly in USA [16] and Europe [17]. very few studies have been conducted in Latin America that identify and describe the characteristics of the people attending these facilities, the pattern of substance use, satisfaction and perceived effectiveness of these treatments by users, and the follow up of the patients who fulfilled the therapeutic plan.

In a previous study conducted in Latin America by the same authors and in collaboration with Latin American Federation of Therapeutic Communities (LAFCT) that identified the TCs, available and their respective quality in Argentina, Brazil, Colombia, Mexico and Peru. Using the database of the available TCs obtained in that study it is intended to identify the sociodemographic variables, consumption patterns, user perception of effectiveness, fulfillment of the treatment plan and follow up of the users in the same 5 countries that provided the database of the TCs.

## Methods

Based on the study conducted in 2013 which identified all the available TCs who accepted to participate and answered a questionnaire about their structure, therapeutic plans and were evaluated according to the De Leon criteria(These are 12 essential items established by a group of authors that should be accomplished in order to deliver quality care to the users) [18, 19]. The TCs that obtained a score equal or higher than 9 points according to De Leon criteria were selected (See Table 1). TCs were excluded if they exclusively worked as an outpatient basis or whose target patients was exclusively minors or took care of fewer than 50 patients or if their therapeutic plan was less than 30 days. Once the sample of TCs that fulfilled the selection criteria and were willing to participate was established, a random number was generated considering a simple distribution. With the subsequent setup of the TCs in a list and then selecting TCs from each country.

**Table 1:** De Leon criteria [18, 19]

Component	Brief description
Planned duration of the treatment	The length of the treatment will be adjusted to the individual needs of each patient.
Alienation from the community	In a residential context, the patients will be kept away from the exterior community 24 h a day for at least some months, before acquiring privileges of permit
Community activities	Excepting the individual counseling, all the activities are scheduled i community with the other residents
Staff roles and functions	Independently of the professional function, each member has to fullfill the function of community member. For this reason, the mission from each member of the staff is to provide help and aids according to the method of community self-help
Residents as role model	The members who show expected conducts and capture the values, ideas and beliefs of the community are used as role model for the other residents
Structured day	The activities are daily planned in order to distract de residents from their cravings, thoughts about consumption and drugs, and also from the routine of their daily living
Job as therapy and education	According to the methodology of self-help, all the members are responsible of the daily management of the facilities. The work is distributed among the users creating responsibilities and duties with education and therapeutic goals
A vision of recovery and right living	There are some established concepts in the methods used by the Therapeutic community to instruct about topics around the rehabilitation and drug cessation through the self-help methodology
Meeting groups between residents	The common sense of conducting this kind of meeting is to create awareness in each patient about patterns and attitudes related to the pattern of consumption that could be identified sharing their experiences with other residents of the community
Awareness training	The main goal of all the therapeutic or educational is to increase self-awareness of the individual about the consequences, impacts and repercussions of their previous conducts and attitudes in themselves and in their social environment.
Personal growth training	To achieve this goal, the community should guarantee education and instructions to the patient of how to identify their own feelings with their respective management and expression in a constructive way and how to share them in community.
Care continuity	Fulfilling the treatment plan, with the goals of increasing self-awareness and change of their vision based in the community and self-help method is the firs step. After this, a network between the user and community should be established in order to keep the process of continuous personal and growth and providing personal experiences to newcomers.

Once selected the TCs in each country, visits were made to each of them for descriptive user surveys on socio-demographic variables, perceived quality and fulfillment of the therapeutic plan of TCs, as well as consumption patterns of patients from the TCs contacted and who were willing to continue participating in the study. The surveys were made by previously trained.

Patients eligible for the survey were those who had entered the TCs early 2009 or late 2012. The questionnaires used were translated to portuguese for the participants of Brazil, and adapted to each of the other countries in order to improve the reception of the user who were willing to participate in the study. These questionnaires were created by the group and were administered by previously trained representatives in each country.

The questions evaluating the perception of TCs from the users were scored from 1 to 5, 1 meaning completely unsatisfied and 5 completely satisfied. For the question of improvement perception the answers were also classified from 1 to 5, being 1 not improvement and 5 totally improvement.

Additionally, a retrospective data gathering was carried out by applying surveys to the TCs directors, requesting information on drop outs, admissions and therapeutic discharges in the three years prior to the study. Except 3 Argentinian TCs that were selected and refused to provide the information requested, the rest did not object to deliver the required information.

After obtaining all patient oral and written informed consent, questionnaires, and having collected the required information from the TCs directors, information was integrated into a Microsoft Excel database. The datasets used and/or analyzed during the current study is available from the corresponding author on reasonable request.

This study was conducted with according to the principles established by the 2000 Helsinki Declaration. The study also counted with the approval of the ethics and review board from each of the Universities or Institutions involved in the study.

## Results

### Therapeutic communities selected and number of participant users

In all, 58 TCs, with 1414 users were interviewed, though much of the information from users of TCs in Argentina was not complete and therefore not included in some analysis of sample data.

The country that provided more participant TCs was Brazil with 20 communities (34%), and less involved was Colombia with only 7 TCs (12%). Most people interviewed were men, accounting for 92% of the surveyed sample, the most common marital status was single, accounting for 62% of the sample. Regarding employment status, 53% reported being employees prior to his admission to the TC, and 46% were unemployed. (See Table 2).

**Table 2:** Number of TCs surveyed by country and sociodemographic characteristics

	n	%
Participant TCs by country		
□ Argentina	9	16
□ Brazil	20	34
□ Colombia	7	12
□ México	10	17
□ Perú	12	21
Interviewed users by country		
□ Argentina	361	26
□ Brazil	300	21
□ Colombia	178	13
□ México	300	21
□ Perú	275	19
Gender		
□ Males	1298	92
□ Females	116	8
Marital Status		
□ Single	673	64
□ Married	168	16
□ Divorced	95	9
□ Widower	11	1
□ Free Union	95	9
□ No Data	11	1
Previous Work Activity^a^		
□ Employee	558	53
□ Unemployed	484	46
□ No Data	11	1

^a^Total number of patients was 1053, there was no data from Argentina

### Consumer age and previous hospitalization

The age of first use of any non-alcohol PSs varied greatly across countries, being the lowest Mexico with first intake made at 4 years of age. As for the older age of first use of a substance, it was reported in Brazil with a report of 53 years of age. Unlike the consumption of younger and older age, the average age was very similar between different countries, generally averages between 15 and 16 years of age (see Table 3).

**Table 3:** Age at first use of substances

	Brazil	Colombia	México	Perú	Total
Age^a^	n	n	n	n	n
Minimum	5	7	4	8	6
Maximum	53	45	46	48	48
Average	16,1	16	15	16	16

^a^There was no data from Argentina

In terms of number of previous hospitalizations, 684 users (65%) reported not having been previously hospitalized, 369 users (35%) reported having been hospitalized at least once before, and 295 (28%) responded that they had been interned three times before their current treatment.

### Substance use

**Fig. 1:**
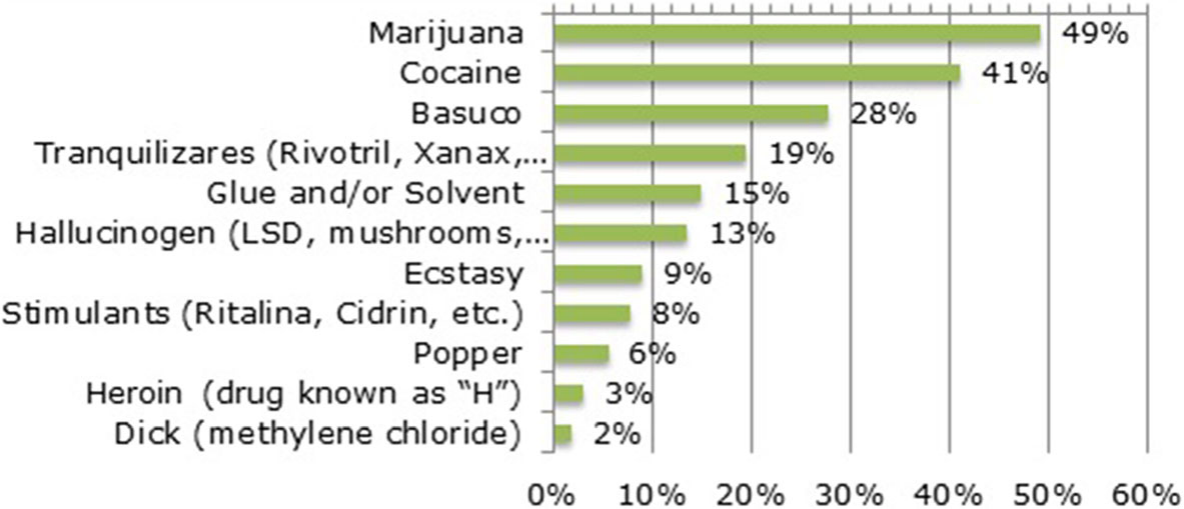
Most consumed substances in the participating countries except Argentina

Users answered if they had consumed some substance different from alcohol in the 30 days prior to their stay in the TC, and identify the non-alcohol substance used. The results across countries are similar, being marijuana the most frequently (49%), followed by cocaine (41%) and basuco (cocaine base paste, possibly mixed with unknown components) users (28%). Among the substances less reported are heroin powder (3%) and dick (2%) (Liquid used for cleaning machines). (See Fig. 1).

Also inquiring among users of alcohol consumption in the last six months, asking about how many times in that period, if had consumed more than 4 drinks if it was a woman, or more than 5 if it was male, in one day. 21% of respondents said no, 18% once, 60% more than once and 1% of respondents did not answer the question.

### User satisfaction survey

The user satisfaction questionnaire evaluated several areas, such as admission, information on the TCs’ internal rules, satisfaction with institution staff, as well as the improvement perception.

The first aspect evaluated (including reports from Argentinian TCs), was the complexity of TCs’ admission process by patient, which according to most respondents (76%) were slightly or not complicated. In addition, 88% of respondents reported that the staff explained to them in detail the institution rules and 68% of users reported being satisfied with the attention time spent by medical personnel (See Table 4). The following data (not including reports from Argentina) provides information about satisfaction with TCs’ staff, including psychiatrists, psychologists, nurses and other personnel. 76% were satisfied with the staff’s work at the institution.

**Table 4:** User satisfaction

	N	%
Admission Process		
□ Not Complicated	785	56
□ Slightly Complicated	287	20
□ Very Complicated	64	4
□ Quite Complicated	50	4
□ Have not participated in admission process	228	16
Time spent by the doctor		
□ Enough	959	68%
□ Not Enough	455	32%
Information on the TCs’ internal rules		
□ Yes	1258	89%
□ No	156	11%

Less than 2% of users interviewed perceived little or no improvement in their underlying conditions, this being a constant in most countries. Most TCs users perceived improvement in their substance use problem, represented by 54% of respondents, showing that the users that participated in the study were perceiving some kind of improvement of their drug addiction without the use of other therapies like drug replacement therapy or an intense psychiatric management (see Table 5).

**Table 5:** Improvement perception reported by users

	Brazil		Colombia		México		Perú		Total	
	n	%	n	%	n	%	n	%	n	%
Totally	83	29	68	38	52	16	55	20,0	258	25
Quite	153	53,5	85	48	195	59,8	139	50,6	572	54
Some	32	11,2	23	13	71	21,8	58	21,0	184	17
A Little	13	4,5	2	1	5	1,5	6	2,2	26	2
Not	5	1,7	0	0	3	0,9	6	2,0	14	1

There was no data from Argentina

### TCs essential elements

Compliance with quality indicators and essential elements in the sample selected of TCs were evaluated, which included “organized and standardized treatment programs”, “family involvement in the treatment plan”, “self-assessments of the therapeutic team”, etc. (Other evaluated indicators, see Table 6). Most managers reported meeting almost all the criteria, however, the only country where all interviewed TCs met 100% of the items was Mexico. In Colombia, the least met indicators were “work as part of the therapeutic program” (71%) and “users are encouraged to “act as if” to develop a more positive attitude” (71%).

**Table 6:** Indicators compliance by the TCs

	Argentina		Brazil		Colombia		México		Perú		Total	
	n	%	n	%	n	%	n	%	n	%	n	%
The therapeutic program includes training in personal decision-making and social skills	9	100	20	100	7	100	10	100	8	67	54	92
Recovery means for TC development of personal identity and global change lifestyle	9	100	20	100	7	100	10	100	12	100	59	100
Users learn conflict resolution skills	9	100	20	100	7	100	10	100	11	92	58	98
The work is part of the therapeutic program	8	88	20	100	5	71	10	100	12	100	55	93
Users are encouraged to “act as if” to develop a more positive attitude	9	100	19	95	5	71	10	100	11	92	55	93
Regularly seminars are held to help residents find a balance between the emotional and cognitive experiences of the TC program	8	88	19	95	6	86	10	100	12	100	56	95
Requesting professional qualifications to former addicts	8	88	10	50	6	86	10	100	11	92	45	76
Advisory services are provided to the user’s family	9	100	20	100	7	100	10	100	12	100	59	100
The TC has medical records and individual records for monitoring and continuous evaluation of services	9	100	19	95	7	100	10	100	12	100	58	98
The TC has guidelines for developing an institutional climate of trust and mutual support; It has a written declaration of user’s rights and duties	9	100	20	100	7	100	10	100	12	100	59	100
There is a flowchart of the TC’s staff functions, which are known and accepted by all	9	100	20	100	7	100	10	100	12	100	59	100
The TC periodically performs an assessment of its effectiveness and efficiency, which includes user’s review and satisfaction	8	88	19	95	7	100	10	100	9	75	53	89

In Brazil, the element with the lowest compliance was “requesting professional qualifications to former addicts" this mean that if a former addict wanted to be an employ of the therapeutic community, he was not asked about any degree or professional qualification in order to be a worker in the TC.In Peru, the lowest score was the criterion of "The therapeutic program includes training in personal decision-making and social skills”. In Argentina, despite the quality indicators were not met by all communities, there was no indicator to be less satisfied compared to the rest.

### Retrospective data collection

Data and medical records of the TCs who agreed to participate (including details of admission and discharge of patients in the time period between the second half of 2009 and the first six months of 2012) were gathered. Should be clear that in this retrospective collection, data from the Argentinian participating institutions was not obtained. Records of 3461 patients were obtained, of which 87% were male and 13% female. The ages of the individuals had a wide range between 13 to 79 years, with an average of 30 years, showing the variability of people who suffer from drug addictions.

**Fig. 2:**
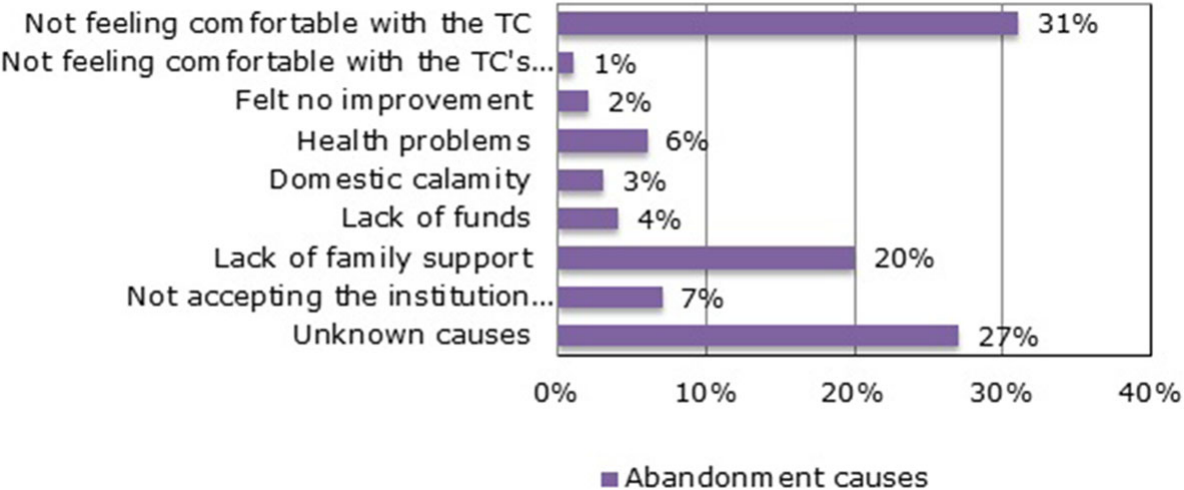
Abandonment reasons

The main reasons for abandonment were collected. A total of 1453 records(42%) were analyzed, in which the most frequent were “not feeling comfortable with the institution” (31%), followed by “ignorance of the cause” (27%) and “lack of family support” (20%). Less frequent reasons were “not feeling comfortable with the institution’s staff” (1%) and “have not perceived any improvement” (2%) (See Fig. 2).

**Fig. 3:**
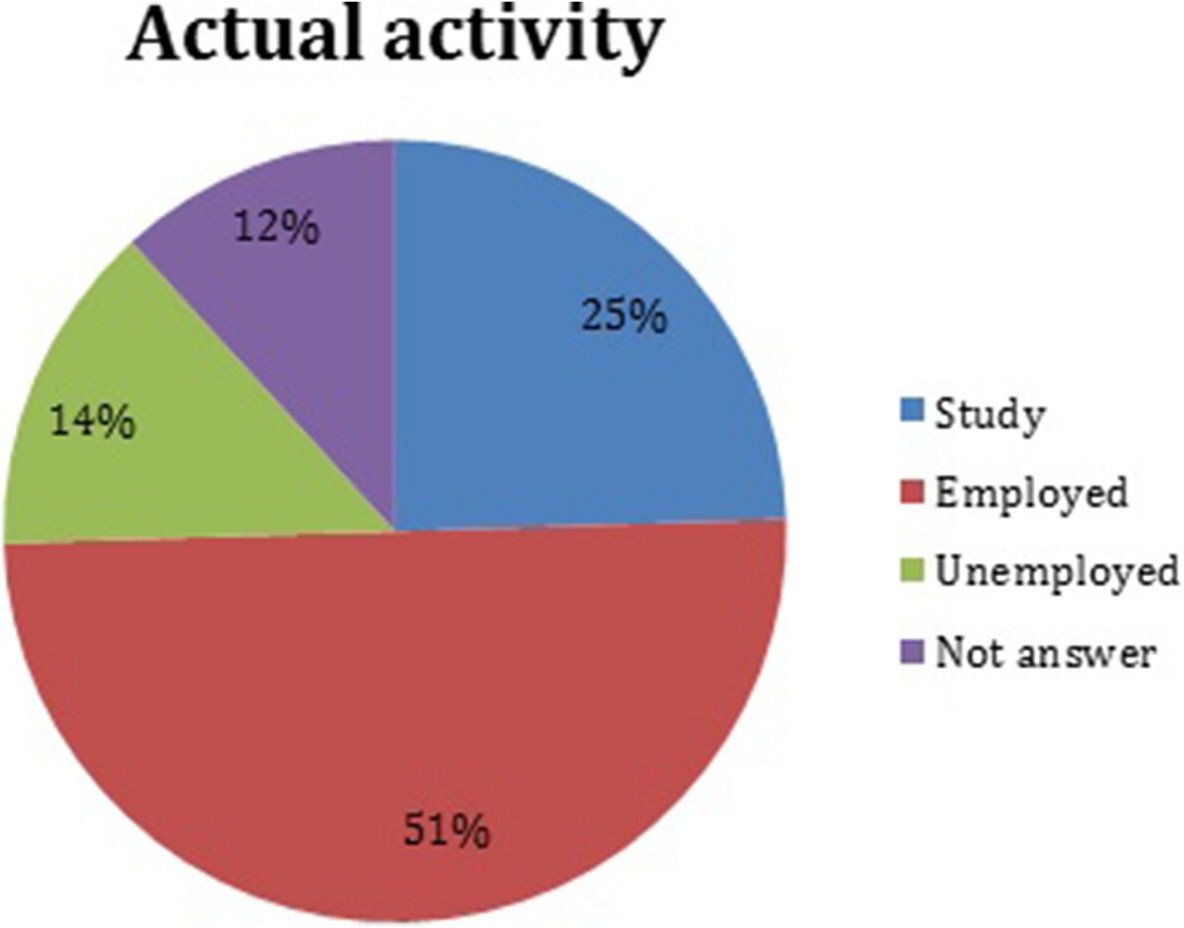
Actual activities of the user who ended their treatment plan

To analyze the reasons for patients discharge, 3461 individuals’ data was obtained, of which 44% were therapeutic discharge, i.e. completion of the treatment plan, and 42% withdrew. For the remaining 14%, no data was obtained. Finally, follow-up at one year of 795 patients main activity was collected, of which the vast majority (51%) was working, 25% were studying, 14% were unemployed, 10% did not know and 2% do not have the data (Fig. 3). The vast majority of these (71%) were conducted by telephone communication, followed by visiting the TCs (23%) and the remainder is distributed among contact via e-mail or home visit.

## Discussion

The findings of this study are the first in Latin America allowing adequate description of the TCs in our region with a high amount of the user reporting a quite to totally improvement just with this intervention . It is the first study in this part of the world, and including five countries allows proper identification of these institutions and regulates in a more uniform manner its existence. This could help to the establishment of more rules and guidelines to unify and standardized the care delivered by this type of institutions.

One of the first findings in our study was that up to 90% of the respondents were men, which may be because the male are more likely to consume PSs compared with women, as issued in 2014 by the UNODC report [20]. Additionally, in a study conducted by Johnson et al. in Thailand, in which they evaluated 22 TCs, most users were men [21]. The sexism or Macho culture spread through all Latin America also could explain the greater proportion of male assisting to this communities because of the Taboo or bad image that this can cause in a woman.

Another finding of our study was that most users of the TCs interviewed were single before admission to the program. These findings are similar to those found by studies such as Fernández- Montalvo [22], in which 72% of respondents were single, while in other studies such as Sadir et al. [23] only 48% were single. In our study, the majority of respondents (53%) were employed before entering the institution, however, reports of employment status among TCs users are highly variable with employment rates of only 17% in one TC in Australia [24], or as high as 64% in Iran . One explanation of these differences may be that in the countries were the study was conducted, most of the TCs are private or belong to a religion and not public and supported by the government. This would make the entrance to this center more difficult because some patients do not have the money or resources to access them.

The average age of first use of a non-alcohol psychoactive substance was 16.1 on average for the entire sample, a value that is similar to those found in other studies for different countries in the region [25] and as reported by the UNODC [20]. Most patients in our sample (68%) had not reported any previous attempt of entry into a TC, and only 28% reported to have entered more than three times; these figures differ from those reported by a study in Iran [23] in which only 25% of respondents had less than two entry attempts to a TC facility and 75% had three or more attempts of entering TCs for treatment. Another study conducted by Darke [24] and colleagues found that up to 58% of patients interviewed had been admitted at least once in a TC treatment .

The non-alcohol substance most frequently consumed in the last 30 days was marijuana, followed by cocaine and basuco (cocaine base), but as mentioned earlier in this article, the epidemiology of substances consumed varies depending on the region. In a study in Thailand, the substances most frequently consumed in the same period were stimulants (ecstasy, speed, ice), followed by inhalants and marijuana [21]. The study by Darke [24] et al., found that the most commonly used drugs in the last six months were benzodiazepines, followed by marijuana and heroin.

The difficulty of getting admission of patients to the TCs was evaluated by patients as slightly or not complicated showing that the main reasons for not using this kind of treatment for substance use are not related to the procedure related to the admission to the community; these results were similar to those found in the study of López- Goñi et al. [26], who found that most of the interviewees reported that the entrance to the TCs was simple. Another aspect evaluated that was not seen in other references, was the user satisfaction with medical care and the health team. 68% of users are satisfied with the care given time, this may be because the patients who accept to go to the TCs hope that if they are going to be intern in a drug-free residence their health should be more supervised than in other places.

Up to 79% of respondents perceived that they had had enough or complete improvement in their condition with the entrance to the TC, and less than 2% did not feel any improvement. These findings are different from those reported by Fernandez et al. [22], who found that only 54% of users had perceived some degree of improvement and 46% had not felt any improvement or deterioration in his health. These could be explained by differences in the users or the number of sample that had each of the studies. Other reasons may be how the improvement was evaluated if just by asking the patient or following them until they relapse or how long was the period of abstinence. These differences mainly exist because there are no standardized measures of treatment success of substance use which could limit the comparisons between the results of different studies about management of drug addictions. Another explanation could be the selection bias because only users who were active in the program were interviewed, but not those who abandoned the program. All these explanations could modify the proportion of users feeling any improvement in their condition.

Evaluation of TCs’ quality indicators evidenced that Latin America meet most of the criteria, however, the criteria “requesting professional qualifications to former addicts” and “use the work as part of the treatment plan”, were lower compliance indicators. Although there are no studies that have used the same criteria we employed, there is another tool to assess the quality of the TCs, known as SEEQ. This questionnaire contains 139 questions and it is narrowed to evaluated 6 dimensions of the TCs in a likert-type questions from 1(very little importance) to 5 (extremely important) and is directed to the directors and staff of the community [27].

SEEQ was used in the Goethal et al. [17]study, in which traditional and modified TCs were evaluated in Europe and USA. Among the findings of this study they come upon that the criteria elements with lower scores in European TCs were “process monitoring and evaluation of its programs”, and “ranking TCs staff members using an established organization chart”. As for the US TCs, the worst scores were achieved in the fields of confrontation and psychological problems arising from drug use as well as family integration in user’s treatment.

The proportion of therapeutic discharges and abandonments are very similar (44% and 42%), these data being different to studies in Australia [24] and Thailand [21], where 17% and 33% of departures ended in abandonment and 34% and 66% in therapeutic discharges respectively. The main reasons for abandonment in our study were “not feeling satisfied with the institution”, “unknown reason” and “lack of family support”, being compared with another study by López- Goñi et al. [26] agrees that the single most frequent reason for abandonment is “not feeling comfortable with the institution”. This finding points out a key question and problem of the TCs designs and how its principles are being adopted by different institutions to make comfortable their users. This issue could be starting point in the design and conduction of new studies to reduce this percentage of users not feeling comfortable with the TCs.

Close monitoring to some users, found that most of the patients were working or studying, and their activities were unknown in 10% of this sub-sample. These findings were also found in a longitudinal study in Spain [22], in which it was found that most users of the evaluated TC, whether they had completed treatment or not, were working. Despite these results, we can not generalize it because of the small sub sample of user that were followed that represented less than 50% of the evaluated users.

The collaboration of several Latin American countries, along with the large sample collected of TCs and users, are the greatest strengths of our study, allowing to find the differences between the TCs available in the countries, the most consumed substances, the consumption onset age and other variables that allow to find out the quality of TCs among the studied countries and an estimate of the services available in our region. Most of the finding of our study are consistent with previous reports of the literature, but with the findings of the high satisfaction rate and the fulfillment of the essential criteria of the TCs it opens new doors in order to propose this method as an alternative for the management of drug addictions.

One of the weaknesses, was selecting centers that have fulfilled a minimum score in the first phase of the study, because this could have biased the interpretation of variables as the fulfillment of essential elements of quality, as may have limited the selection of center users to those with better qualifications, thereby reducing the possibility of extrapolating the findings of our study. Another major limitation of both the study and participating centers was the high proportion of missing information on variables as the length of stay of the patients, “reasons for abandonment”, monitoring and discharges during the period in which the study was conducted. Selection bias could have modified the results because only active users were interviewed for the satisfaction and improvement status, but users who already fulfilled their treatment plan were not contacted.

## Conclusion

TC’s are one of the therapeutic options for the treatment of patients with substance use disorder, so the findings of this study showed that the participating TCs fulfilled the minimum quality criteria to provide management to the patients and most of the patients are satisfied with the received treatment. Despite, there is still room to improve, especially in the follow up of the patients in order to provide more complete data about the effectiveness of this interventions in the patient in Latin America.TCs are also widespread in the region and the knowledge of how are they performing and their quality may allow them to be more popular among the people of the region and the governments.

## Abbreviations

HIV: Human Immunodeficiency Virus
LAFCT: Latin American Federation of Therapeutic Communities
NMHS: National Mental Health Survey
PSs: Psychoactive substances
TCs: Therapeutic Communities
UNODC: United Nations Office on Drugs and Crime
WHO: World Health Organization
YLL: Years of life lost
